# The struggle of preserving self-esteem and professional identity: A grounded theory study of specialists’ experiences serving in the public health system in Rajasthan, India

**DOI:** 10.1371/journal.pone.0325820

**Published:** 2025-06-18

**Authors:** Anushree Joshi, Jallavi Panchamia, Dileep Mavalankar, Bharati Sharma

**Affiliations:** Department of Health Policy, Management and Behavioural Science, Indian Institute of Public Health Gandhinagar, IIPHG; CMC Vellore, IPH India, INDIA

## Abstract

This study aimed to explore and describe the experiences of specialist physicians at rural community health centers (CHCs) and determine the factors accounting for their retention and attrition in Rajasthan, India. Twenty-one medical professionals from different public health facilities in Rajasthan, selected through purposive sampling, were interviewed in depth. Strauss and Corbin’s grounded theory approach was used to develop a theory/model. Open, axial and selective coding were used to identify the major themes/categories and develop the core category of the model. Strauss and Corbin’s paradigm model was employed to establish linkages among the categories structured in terms of conditions, action-interaction strategies and consequences to explore the experiences of specialist physicians. The central theme of the model, *‘The struggle of preserving Self-esteem and Professional identity’,* captures the experiences of specialist physicians working at the rural CHCs in Rajasthan’s public healthcare system. The disadvantages of an underdeveloped rural setting, local political climate and various healthcare system factors challenge the specialists’ self-esteem and professional identity culminating in their decision to remain or quit the system. This further leads to their acute shortage in the system which affects the quality and provision of rural healthcare services in the state. The state government and essential stakeholders must collaborate to enact policies addressing specialist physicians’ personal and professional needs, which include improving developmental infrastructure, healthcare system processes, local governance and accountability. Such policy packages can generate sustainable solutions to mitigate the high attrition rate of specialists in rural CHCs and strengthen rural healthcare services in Rajasthan.

## Introduction

India is one of the 57 countries listed as having an acute shortage of human resources for health (HRH) [1]. India ranks 154^th^ amongst 194 countries in terms of the healthcare access and quality index [2]. According to the National Sample Survey Organization survey 2017–2018 [3] and WHO’s National Health Workforce Accounts portal for 2024 [4], India’s physician density is as low as 6.1 and 7.27 physicians per 10,000 people, respectively [3,4]. This density is far below the WHO’s current threshold of 44.5 health workers per 10,000 people [5] and necessitates policy attention.

Attaining Universal health coverage (UHC) goals related to health and well-being has substantial financial and policy implications due to the shortage of healthcare professionals, especially specialist physicians [6]. Specialist physicians (or specialists) refer to those health professionals who possess post-graduate (PG) education in clinical disciplines at rural community health centers (CHCs) in India [1,6]. Rural CHCs are 30-bed block-level medical facilities offering specialized care in medicine, obstetrics and gynecology (OB-GYN), surgery, and pediatrics [7]. As per the Dynamics of India (Infrastructure and Human Resources) 2022–2023 report, India’s public health sector is currently facing a critical shortage (79.5%) of specialist physicians in rural CHCs [7]. Only 913 of India’s 5,491 functioning rural CHCs have specialists in the four major specialties—medicine, surgery, pediatrics, OB-GYN—as required, which calls for immediate policy intervention [7]. Table 1 below provides detailed statistics on the vacancies of specialists at rural CHCs in India.

**Table 1:** Vacancies of specialist physicians at rural CHCs.

Specialist physician category	Vacancy rate (%) (March 2023)	Number of vacancies (March 2023)
Medicine	69.2	1512
Surgery	83.2	1824
Pediatrics	67.1	1456
Obstetrics and Gynecology	74.2	1628

Source: Health Dynamics of India (Infrastructure and Human Resources) 2022–2023 Report.

Various reasons, such as inadequate remuneration, delayed promotions, lack of entry cadre for specialists [8], ineffective policy initiatives [6], restricted scope of professional advancement prospects and availability of essential medical resources within the healthcare system account for the shortage of specialists in the Indian public health sector [1,9–12]. Consequently, the deficiency of specialists results in an increased disease burden and mortality among the rural population [13,14}. It also forces rural people to seek specialized services from higher-level health facilities, contributing to their higher monetary load and untimely deaths [15].

To enhance their retention, the Ministry of Health and Family Welfare and state governments have recently executed numerous programs throughout various Indian states [16–18]. Retention approaches have included mandatory rural duties, offering specific financial incentives and linking PG program admission to rural jobs [16–18]. Despite these efforts, creating effective strategies for retaining specialists in rural areas remains challenging, which impacts service availability and affordability to achieve UHC goals [1,6,19]. Therefore, ensuring that all CHCs are adequately equipped and staffed with a team of four specialists is essential, as required by the Indian Public Health Standards of the Government of India [20].

There is also sex segregation among physicians in India. Most physicians are males, while the majority of midwives and nurses are females [3,19]. Only 14.2% of physicians in India are females [4]. This underrepresentation of female physicians in the health system may have a greater impact on women’s access to healthcare facilities than any other factor [13,14].

In the past, research evidence on the rural healthcare workforce shortage in India predominantly comes from general physicians, nurses and midwives, highlighting a significant knowledge gap in adequately exploring the experiences of specialists working in rural regions [13,14,21–23]. Many previous studies on specialist shortages have been reported from developed nations [24–26]. Additionally, a dearth of literature exists in the Indian context and other lower- and middle-income countries (LMICs) specifically on acquiring a more thorough comprehension of the evidence-based human resources (HR) strategies necessary to address rural attrition issues of specialists [27]. Therefore, given the current rise in inequities in the Indian rural healthcare system, exploring their experiences is essential to understanding the factors contributing to their lower retention and higher turnover rates. This is vital to improving rural healthcare services and developing effective HR strategies [1,6,17]. To comprehend this lack of availability of specialists and their asymmetrical gender distribution, it is imperative to explore the context and the job factors in terms of their experiences which influence their decision to quit or work at rural CHCs.

This study is a part of the first author’s doctoral project, which used a mixed methodology to identify essential job attributes valued by specialists to work in rural CHCs of Rajasthan using the Discrete Choice Experiment (DCE) method. DCE is a quantitative technique for assessing the stated preferences of health workers for a job as a function of the job’s characteristics [28,29]. This qualitative study aimed to identify indicators for designing the DCE through in-depth interviews with specialists. Besides helping with the identification of the indicators for DCE, the current study has two primary research objectives: First, to explore and describe the factors and circumstances from their rich life experiences that influence and shape their preferences in working at rural CHCs in Rajasthan, one of the largest Indian states in terms of geographical area [30]. Second, to comprehend the factors contributing to their gender disparity at rural CHCs. These experiences will help deepen our understanding of the underlying factors influencing their motivation to continue or quit the public health system. Therefore, this qualitative study explores the reasons for this long-standing acute shortage of specialists in rural CHCs in Rajasthan.

## Methodology

### Study setting

Rajasthan is the largest Indian state, covering 10.4% of the country’s area in the western region. It is also the seventh-largest Indian state by population and is divided into 41 districts [30]. Rajasthan, with a population of 68.6 million, constitutes 5.7% of India’s total population [30].

**Fig 1:**
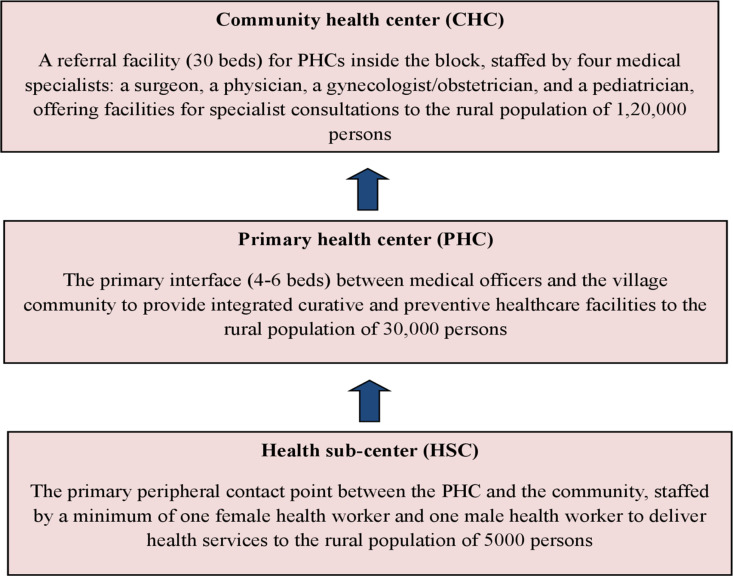
A figure depicting the Indian rural healthcare system.

In rural India, Government health services are primarily delivered through a three-tiered structure of publicly funded health facilities, comprising CHCs – 24/7 rural hospitals catering to approximately 120,000 individuals – at the apex, primary health centers (PHCs) in the intermediate tier and health sub-centers (HSCs) at the base. Rajasthan’s rural healthcare infrastructure follows the national norms of the three-tier system consisting of 14042 HSCs, 2179 PHCs and 650 CHCs [7] (Fig 1).

Compared to the necessary 2600 specialist physicians required for the 650 operational CHCs in Rajasthan, there is still a shortfall of 2090 specialists [7]. There are still 650 physicians, 650 surgeons, 650 OB-GYNs and 650 pediatricians needed in the state’s rural CHCs [7]. Compared to other Indian states and the national average, the state fares poorly regarding health outcomes, mainly due to inadequate workplace infrastructure, inequitable distribution and lack of specialist physicians. Hence, providing specialized health services in rural areas remains a big challenge for the state, requiring immediate policy attention [31–33].

### Study design and data collection

The grounded theory approach developed by Strauss and Corbin [34] was considered appropriate for this study as the aim was to determine the factors influencing the experiences of specialist physicians and their decision to either stay or exit the rural healthcare system, which can be considered a process. This approach was considered suitable for the current study as the goal was to build a model/theory for the process to better understand specialists’ higher attrition rate, structured systematically in terms of certain conditions, actions- interactions and consequences by exploring the underlying factors grounded in data. An interview guide was developed to explore the process in depth. A few exploratory interviews were conducted with key informants for initial sensitizing concepts. Simultaneously, the relevant literature on HRH and a few context-specific policy documents were reviewed. The interview guide included matters related to personal work situations and experiences, professional demands of the workplace, motivation for choosing rural/urban locations, as well as happy and unhappy moments in work life.

Data were collected from September 2021 to December 2021. The first author did the interviews and twenty-one in-depth interviews were conducted via the telephone due to the COVID-19 pandemic. Each interview lasted 40 minutes to one hour and was audio recorded and transcribed verbatim in the vernacular. Analysis began during data collection. During the first round of interviews, two retired specialist physicians with 25–30 years of considerable experience in the rural CHCs of the state were included. Themes related to their professional job demands and the specific incentives needed to go on rural postings emerged from the first round. These themes included – general developmental infrastructure (residential facilities, educational infrastructure, workplace location), local political climate (constant challenge to expertise, HR policies, professional upskilling, reward-performance linkages, medico-legal cases), healthcare system factors (workplace infrastructure, workload level, financial incentives) and non-financial incentives (personal and professional resources). Likewise, the second round comprised interviews with 15 specialists working at rural CHCs and government district hospitals (DHs). DHs are referral centers for CHCs; responsible for delivering advanced healthcare services to the district population [35] and four resident doctors studying for PG medical examinations to confirm and saturate the property-dimensions of the concepts, categories and core category. Themes related to these specialists’ personal and professional motivation to join or exit the rural healthcare system emerged in the second round of analysis. These themes include – extrinsic and intrinsic intermediate sets of consequences (psychological factors), strategies to quit or continue in the system (risk-taking, acceptance and adaptation) and health system consequences (demotivation and shortage of specialist physicians) (S1 Appendix). The interviews continued until theoretical saturation was achieved. To ensure data quality, notes were taken during the interviews and peer debriefing with the research team helped to validate the findings. Member-checking was performed with a few medical health specialists, specialist physicians and medical officers to verify the themes and primary rural retention challenges in real-life situations.

### Participants

Medical residents doing PG specialization and specialist physicians were purposively selected from different districts of Rajasthan for the study, such as Sikar, Jhalawar, Jodhpur, Bikaner, Jaipur, Bhilwara, Jhunjhunu and Nagaur. These districts were selected to capture the variation in geographical area-wise work contexts and related experiences among the participants. The PG medical students were second and third-year residents in medicine, surgery, pediatrics and OB-GYN from various government and private medical colleges in Rajasthan who had experience working in the state’s rural CHC. The specialists from the same specialties were selected from different rural CHCs and state government DHs. The participants from these specialties were selected to understand their experiences as health professionals in the system concerning their acute shortage at the rural CHCs [7,31–33]. The specialists at rural CHCs differed from those at the DHs in their scope of practice, resource availability, patient load and professional growth opportunities. The study also included senior specialist physicians who retired from the state’s rural CHCs. The sample comprised 15 male and 6 female participants, out of which 2 were retired senior specialist physicians who worked in the public health system for the last 25–30 years, 9 were specialists working at rural CHCs, 6 were specialists working at Government DHs and 4 were PG medical residents. The study cohort, from various public health facilities, was considered to gain a broader perspective and wide range of scenarios of the systemic issues and relative challenges related to the state’s rural healthcare system based on their expertise and vast experiences of continuing/exiting the system. Of the participants interviewed, 10 were those who left the rural healthcare system and 11 were those who continued (Table 2).

**Table 2:** Participants’ Background Characteristics.

Variables	Category	Participants (N = 21)
**Gender**	Male Female	15 6
**Age (years)**	Mean Range Modal Age group	37.71 28-71 30 and 38 years
**Job designation**	PG Medical Residents Specialist physicians (Rural CHCs)	4
	(Pediatricians, OB-GYNs, Surgeons and Physicians)	9
	Specialist physicians (Government DHs) (Pediatricians, OB-GYNs, Surgeons and Physicians)	6
	Senior specialist physicians (Retired) (Rural CHCs)	2
**Family Background**	Rural Urban	6 15
**Marital Status**	Married Unmarried	16 5
**Children**	Having Children Not having children	13 8
**Employment status**	Those who left the rural health system	10
	Those who continued to work in the rural health system	11

### Ethical considerations

The research and ethics committee at the Indian Institute of Public Health Gandhinagar, Gujarat approved this study (TRC-IEC No. TRC/2021–22/14). Relevant permission for the study was also taken from the Secretary of the Department of Medical, Health and Family Welfare and the Department of Medical Education, Government of Rajasthan, after approval from the ethics committee at IIPHG. Considering the severity of the COVID–19 pandemic, individual consent forms were shared online and oral consent was taken for permission to participate, audio recordings, transcription of interviews and taking notes. Participants were assured of voluntary participation and the freedom to quit at any time. Their confidentiality was maintained using unique codes that were accessible only to the research team.

### Data analysis

**Fig 2:**
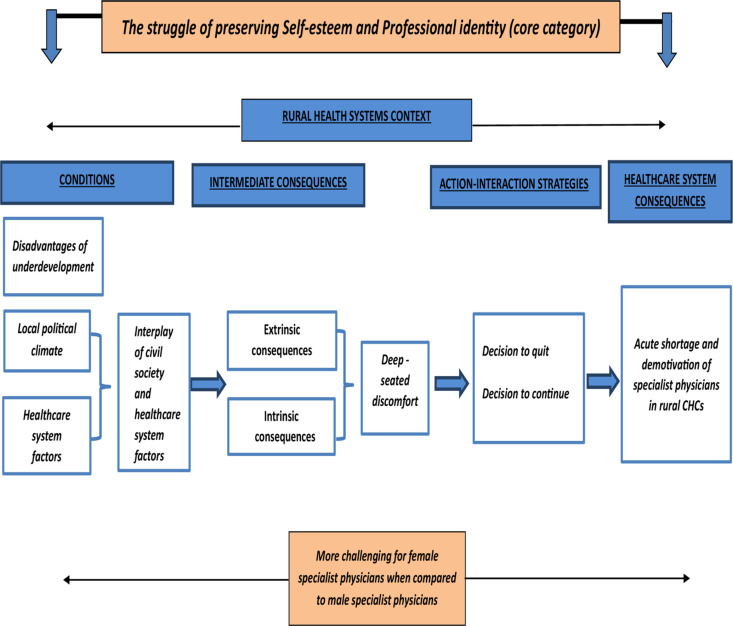
A figure depicting the experiences of specialist physicians working at rural CHCs of Rajasthan.

The paradigm model by Strauss and Corbin [34] was used to construct the grounded theory. The paradigm model is a framework that systematically organizes and delineates the interrelationships among open (basic level concepts), axial (higher-level concepts/categories) and the core category (central theme) to build a theory. The systematic organization of this model aids in delivering a thorough comprehension of the researched phenomena concerning conditions, action-interaction methods and consequences in terms of contextually situated effects. Context encompasses the circumstances/conditions constituting a situation, the interpretations assigned to them (problem or goal), the actions and interactions individuals undertake to attain desired results and the consequences arising from their actions [34]. Open, axial and selective coding was performed for data analysis using ATLAS.ti software (Version 7.5.18) [36] to identify the basic, higher-level concepts and the core category. The codes were clustered separately for all the transcripts and constantly compared across transcripts to identify the major themes emerging for the model. Several categories/themes and sub-categories/sub-themes (axial codes) developed from basic-level concepts (open codes) generated during the data analysis were integrated and linked together to identify the model’s core category (central theme). Once the basic level concepts, categories, sub-categories and the core category were developed, the Paradigm Model of Strauss and Corbin (2015) [34] was used to construct relationships amongst the concepts and categories in terms of conditions, action-interaction strategies and consequences. Simultaneously, memos were written to reflect on the participants’ descriptions and the emotions, meanings and responses they attached to events to develop and formulate the grounded theory. The model was constructed from an analysis of the properties (characteristics) and dimensions (range) of the basic-level concepts, categories and the core category (Fig 2). The core category was identified to be *‘The struggle of preserving Self-esteem and Professional identity. *’**

## Findings

The constructed model is presented in Fig 2 as a complex process depicting the experiences of specialists when working in the rural healthcare system in Rajasthan. The model comprises conditions/circumstances, intermediate consequences, action-interaction strategies and consequences for specialists and the healthcare system. Central to the specialists’ experiences was the struggle to preserve their self-esteem while retaining their professional pride and identity, which they felt was constantly challenged. This was identified as the core category of the model, *‘The struggle of preserving Self-esteem and Professional identity’* (Fig 2). Several categories and sub-categories contributed to this struggle of specialists in the context of rural healthcare systems.

The categories *‘disadvantages of underdevelopment’, ‘local political climate’* and *‘healthcare system factors’* described the conditions influencing specialists’ decisions. Fig 2 shows that a constant interplay between the conditions of *‘civil society’ (local political climate)* and ‘*healthcare system’* leads to *‘extrinsic’* and *‘intrinsic’* sets of intermediate consequences, resulting in *‘deep-seated discomfort.’* This becomes a condition for further action–interaction strategies, culminating in the specialists’ decision to *‘quit’* or *‘continue’* in the system.

Either of these decisions has consequences for the healthcare system (*resurrection of self-esteem versus surrendering and compromising, leading to demotivation and acute shortage of specialist physicians*)*.*

### 1. Conditions/Circumstances

Conditions/circumstances refer to individuals’ perceived justifications for events and the explanations they establish for their actions and interactions [34]. The categories *‘disadvantages of underdevelopment’, ‘local political climate’* and *‘healthcare system factors’* denote the conditions that affect specialists’ decision-making processes.

#### 1.1 The disadvantages of underdevelopment.

Bound within this concept lies the need for more general development of infrastructural facilities in rural areas, which hampers the quality of life for specialists working in rural areas. Specialists working at rural CHCs face significant challenges stemming from inadequate infrastructural development such as residential facilities, educational infrastructure and workplace location. The specialists believed the lack of proper residential facilities interfered with their privacy and lifestyle. The lack of maintenance and essential facilities like stable water and electricity supply at the allotted residential quarters added to their daily burden.

As shared by a *PG medical resident (male, 31 years, Government medical college)*,

*‘The quarters are never good for a specialist to live at the CHC level. Basic facilities are always compromised. There is no electricity facility, water supply or security. This is one of the big reasons for specialists not joining rural jobs!’*

They felt unsafe and vulnerable when impatient villagers demanded services outside their working hours contributing to their struggle to live in these areas with their families. A *specialist physician (male, 40 years, rural CHC)* shared, *‘Some villagers never understand that we have a personal life. They come to seek treatment after we have reached home and our working hours have ended, thinking that being physicians, we are made for them and bound to become available all the time for them. We cannot deny them. Otherwise they create unnecessary trouble for us.’*

Since it conflicted with the specialists’ goals for family life, the lack of educational infrastructure at rural CHCs was found to be a more significant concern among most of them for not joining the rural health centers. Although highly educated and skilled, they shared that they have had to compromise on their children’s educational needs, recreational activities and personality development. This shortcoming becomes a barrier to retaining specialists in rural health centers, as it contradicts their dignified lifestyle.

A *specialist physician (male, 35 years, rural CHC)* shared,

*‘Being a specialist physician, you also want a living status for yourself and your family. This is not possible in a rural setting. There is always a lack of good schools and proper recreational facilities for children. There is a lack of other basic facilities that one cannot compromise.’*

Additionally, far-away workplace locations from one’s hometowns, family concerns and inadequate transportation facilities in rural settings significantly hinder specialists from reaching the health centers in time. They felt that job postings were hardly assigned to them near their hometowns, which made them unwilling to join and continue their jobs in rural areas. A *specialist physician (male, 38 years, Government DH)* shared,

*‘What happens is that a specialist is never given a posting in his hometown. He is always placed at locations far away from his hometown. How does continuing with jobs in such a scenario make sense? A person has a family life also.’*

Such combined infrastructural deficiencies foster an environment where specialists struggle to reconcile personal and professional responsibilities, exacerbating systemic dissatisfaction with rural healthcare assignments and making rural healthcare postings an unappealing choice for themselves.

#### 1.2 Local political climate.

The *‘local political climate’* category reflects the work environment of rural CHCs, which specialists perceive as being shrouded in power dynamics of non-technical people such as the village Sarpanch and members of the legislative assembly (MLAs). Such an environment is characterized by a lack of formal operational mechanisms and a socio-economic-political backdrop. A Sarpanch is an elected decision-maker inside the village-level constitutional entity known as the Gram Sabha in India [37]. An MLA is an individual elected by the constituents of an electoral district to serve in the state legislature under the Indian system of Government [38]. Panchayati Raj Institution (PRI) is a system of local self-governance for villages in rural India, which seeks to implement local democracy and promote development in rural regions [37]. As per the Panchayati Raj System, which was introduced nationally in the year 1993, the village head Sarpanch and other elected members at the district level have the right to monitor and demand quality health services as part of empowering the communities and improving accountability of the healthcare system [37]. However, the specialists were firmly against the excessive involvement and escalation of power to these people to constantly monitor and govern them despite being untrained in the functionality of the healthcare system. According to the specialists, as elucidated below, the involvement of such local governing authorities has disrupted their internal accountability, professional autonomy and identity.

*A specialist physician (male, 36 years, rural CHC)* shared,

*‘Sarpanch thinks that everything is under their control and interferes in everything we do. They will even check our attendance register and inspect everything about who is coming, who is going, and day-to-day work-related activities, despite not knowing the medical health system.’*

Likewise, specialists perceived their transfers as unjustified without a well-defined transfer policy. They found transfer policies strictly politically driven, which lacked procedural fairness and rationalization in their execution. Only those with firm political connections enjoyed the perks of getting their desired rural postings in the system, while the rest struggled to survive. Firm political grounding coupled with poor execution of HR policies contributed to their low morale, inadequate geographical distribution and migration.

A *specialist physician (male, 37 years, Government DH)* quipped, *‘There is no policy like a transfer policy. They can post anyone wherever they want to post them.’*

Similarly, promotions in the healthcare system comprised elements of *‘political unfairness’* as they favored specific groups of people and offered specialists limited room to shape their career prospects and build their capacity. Specialists shared that promotions to senior authority positions, such as chief medical and health officer (CMHO) and block medical and health officer, were not rationalized and lacked appropriate evaluation channels, which lowered their work satisfaction.

A *senior specialist physician (male, 68 years, retired)* claimed,

*‘Now, the entire setup is political. If a specialist is politically powerful, he easily becomes CMHO.’*

Meanwhile, due to the lack of capacity-building workshops at rural CHCs, specialists also felt the challenge of their professional upskilling. They further expressed that due to systemic pitfalls, they could not use their professional clinical competence, making them feel like program managers rather than specialist clinicians with specific medical skills. They started feeling a never-ending juxtaposition between their clinical roles (which they were trained for) and their managerial roles (which they were expected to perform) to survive and serve the authorities’ ulterior motives. Specialists were also uncomfortable knowing that the system worked in a way that made them feel used and taken advantage of. A *specialist physician (female, 34 years, rural CHC) added,*

*‘Training is never related to getting acquainted with new methods or for our capacity building, but it is always related to launching new programs, which is useless to us as specialists.’*

Rather than systematic and transparent evaluation procedures, practices focusing on managing health program implementation (number of immunizations administered, diagnostic tests conducted, sterilizations performed and other related follow-up activities) instead of the health-related clinical outcomes were used to assess the performance of specialists. Such performance evaluation procedures created a sense of discontent among the specialists.

A *PG medical resident (male, 34 years, Private medical college)* shared, *‘There is a monthly system for providing performance feedback to us, but unfortunately, the feedback is not about the clinical treatments we do; rather, it is based on our clerical activities.’*

At the same time, specialists perceived certain healthcare system practices, such as medico-legal cases as morally corrupt and unethical. Medico-legal issues refer to medical and clinical cases with legal implications [39]. They found this strictly unethical due to a lack of autonomy to exercise their professional rights. Ethical issues in medico-legal cases and diminished autonomy in decision-making intensified feelings of moral conflict among the specialists, which was evident in the narrative given by one of the *specialist physicians (male, 37 years, rural CHC)*, wherein he was asked to fabricate a patient’s clinical report to help the patient’s family claim financial benefits from targeted government health insurance schemes. Concerned about his job security, he felt powerless when one of the local political figures asked for an unwarranted favor because his refusal could have adversely affected his future professional prospects. According to him,

*‘If someone from a rich family expires in the village, then a specialist is pressurized to make false postmortem reports for his death. Maybe that person died due to a heart attack, but we are forced to make a false document for that person, stating that he died due to falling from a height.’*

The same participant added*, ‘A specialist is supposed to sign fake documents for a patient’s family, and if he denies it, he gets a call from some minister. Moreover, he must sign; otherwise, he gets his transfer the next day.’*

Such a local political climate involving systemic deficiencies at multiple ladders at rural health centers undermined specialists’ ability to uphold their professional identities and self-esteem, leaving them undervalued, underutilized and disconnected from their intrinsic roles as specialist healthcare providers.

#### 1.3 Healthcare system factors.

*The ‘healthcare system factors’* category refers to the working conditions and hierarchical organization structure and processes at rural CHCs. The specialists perceived the workplace infrastructure of rural CHCs as limiting their ability to utilize their specialized skills, as there was often a scarcity of proper medical equipments, functional operation theatres, blood banks and essential facilities like water and electricity. The rigid allocation of resources did not cater to patients’ needs limiting specialists’ ability to provide care; for instance- specialists were compelled to prescribe drugs that were in supply rather than those needed by patients.

A *specialist physician (male, 39 years, rural CHC)* shared, *‘There are no medicines available that we want to give to the patients, no facilities, no investigations, and no instruments we want. There is no proper availability of water and electricity in rural CHCs. Even blood banks are far away, making patient care difficult. Unfortunately, we are forced to prescribe specific drugs that are in current supply, even if they are not adequate for the patients.’*

Despite being competent enough to perform advanced investigations or surgeries, the specialists felt like birds without wings as they only had the facilities to perform simple surgeries and refrained from taking complicated medical cases.

*A PG medical resident (female, 34 years, Private medical college)* added, *‘Machines need to be better in terms of their functioning ability; they always have some technical errors. Moreover, if they will not function, how will we function? So, we refer patients to higher health facilities despite knowing we can treat them.’*

Specialists further stated that the burden of participating in multiple meetings at the block and district levels shifted the focus of those in charge of the system from patient care to implementing public health management programs. Such non-clinical administrative tasks used to pile up their workload beyond their sanctioned working hours. Staying at the rural CHCs after working hours due to the constant influx of patients made them feel overburdened in the absence of other specialists and subordinate staff.

*According to a specialist physician (male, 35 years, rural CHC),*

*‘We have to perform all the tasks except treating the patients. We are never asked about how many patients we treat and how many we refer to the next level. Instead, we must update the system about implementing different health programs. Because of such things, we are always busy making and sending the reports.’*

Despite their desire to concentrate on providing patient care, HR limitations reduced their ability to meet these expectations. Another *specialist physician (male, 38 years, rural CHC)* shared,

*‘The whole subordinate staff is untrained. They do not even know the basic procedures to perform. We have to tell them everything. We do not have a team of other specialist physicians, like anesthesia, without whom we cannot perform surgeries effectively.’*

Monetary incentivization was the major motivation for the specialists to accept rural postings. However, they soon desired salary increments after realizing the working conditions were inconsistent with their work parameters and career standards.

One *specialist physician (female, 35 years, rural CHC)* expressed, *‘Salary is the only and one of the major things that motivates us to join rural jobs. But still, we feel unsatisfied because nothing, like work conditions, infrastructure and living conditions, aligns with what we need to work at the rural CHCs.’*

***Complex interplay of civil society and healthcare system factors:*** In the rural healthcare system context, the complex interplay of civil society and healthcare system depicts the intricate accountability mechanisms of relationships among local governing bodies, senior administrative officials, rural communities and specialists in rural CHCs. The extent to which specialists can effectively exercise their professional autonomy to provide health services in the rural sector depends mainly on the interplay between civil society and the healthcare system. Such an interplay highlights how the current healthcare system, rural communities, combined ownership of local governing bodies and senior administrative officials have interfered with specialists’ professional autonomy and competence in the health system over time. This interplay is driven by resource scarcity, skewed power relations and a lack of sovereignty to exercise accountability. These factors impact their overt and covert experiences influencing their self-esteem, self-perceptions and professional identity. It leads to two intermediate consequences: extrinsic, including power struggles with senior administrative officials and local governing bodies, uneasy patient relations; intrinsic, which includes compromising with deskilling and self-role distance.

### 2. Intermediate consequences

In the paradigm model, consequences are anticipated or actual outcomes of actions and interactions [34]. The categories *‘extrinsic consequences’* and *‘intrinsic consequences’* portray the intermediate consequences specialists have faced in the rural healthcare system over many years. These consequences resulted from their unpleasant experiences interacting with the governing bodies and local community. They further served as precursor processes that led to major healthcare system consequences.

#### 2.1 Extrinsic consequences.

The persistent struggle for power by the Sarpanch, local political leaders and senior administrative officials limited the work freedom of specialists as reputed health professionals. Despite learning the necessary skills and undertaking advanced training, the specialists reported having no right or say in the system. According to them, the constant burden of maintaining accountability without authority and an authoritative leadership style by non-technical people and senior authorities added to their invisibility in their professional roles. Such power struggles created a sense of entrapment and exasperation among the specialists making them feel caged and powerless.

A *specialist physician (male, 37 years, rural CHC)* shared, *‘The medical exam is one of the toughest exams in the country. Even after learning and practicing so much in the medical field, we still cannot perform our role as we lack work autonomy due to interference of Sarpanch and other people, which makes us feel powerless.’*

According to the specialists, a general mistrust of clinical treatment procedures existed among the rural community members which hindered the establishment of compassionate relationships with the patients. Specialists revealed that they always bore the brunt of the health facilities’ inadequacies while being constantly judged for their actions and poor patient clinical outcomes (which, in reality, result from the lacunae in the system). With this lack of mutual understanding critical to their relationship with patients, they reported that they were never at ease in the community. *A specialist physician (male, 35 years, rural CHC)* stated,

*‘In today’s times, the most important thing to a specialist is saving his life because if something unfortunate happens due to a systemic fault, 15 to 20 people will gather at that place (CHC). So, in that case, I will refer the patient to the next level. Because, unfortunately, if he dies, then I have to face serious consequences.’*

Strong solidarity between the villagers and the local leaders often made the villagers vicious in strategically seeking the community’s attention for political benefits through unnecessary protests. The use of threats and violence during political negotiations indicated the relentless nature of the local communities in demonstrating their control of the health centers. Such malevolence often made specialists vulnerable, with neither their physical nor psychological safety considered a priority. *A specialist physician (male, 36 years, Government DH)* shared,

*‘The more nuisance villagers create, the more attention they will get from the rural public and through such activities, they want to gain fame for their political leaders by highlighting them. I have seen it; physical violence is a huge issue.’*

#### 2.2 Intrinsic consequences.

Specialists working at rural CHCs faced internal psychological struggles that profoundly intensified their struggle in terms of *‘Compromising with deskilling’* and *‘Self-role distance.’*

Compromising with deskilling refers to adjusting to the diminished skill level necessary for accomplishing specific tasks [40]. Specialists had complex reminiscences of their professional experiences. They were tacit in their feelings of being deskilled, pertinently rationalizing it by acknowledging the resource-poor restricted settings of their professional work environment. Although they aspired to provide complete patient care, the absence of workplace infrastructure, work autonomy, essential tools and assistance compelled them to diminish their standards, resulting in feelings of compromising with their deskilling and inadequacy. The identified deskilling outcomes encompassed diminished clinical knowledge, reduced patient trust and lowered confidence in clinical decision-making.

*A PG medical resident (female, 33 years, Government medical college)* shared, *‘We work so hard to enter our professional medical journey. However, soon after being in the system for 3-4 years and facing the realities of our work environment, we realize we have only one option except to accept this scenario, and gradually, we lose touch with our learned skills.’*

Besides this, the experience of self-role distance created an inner battle among the specialists as they felt conflicted between their professional ambitions and the rigid limitations of their work environment. Self-role distance is the psychological disparity between an individual’s professional aspirations and job experiences [41]. The specialists experienced a tangled web of behavioral complexities due to contradictions between their ideal and designated professional roles. Such role ambiguity impeded specialists’ job performance and goal attainment in providing specialized treatment services.

A *specialist physician (male, 36 years, rural CHC),* who is a surgeon by training but could not perform procedures at a rural CHC, shared, *‘There comes the point when we forget who we are! I had never felt so low and helpless in my entire life; despite being a surgeon, I was practicing as a general physician.’*

These extrinsic and intrinsic consequences collectively depict the challenges of specialists in navigating low job satisfaction and increasing emotional disconnection from their professional roles in the rural healthcare system.

***Deep-seated discomfort:*** The blend of intrinsic and extrinsic self-consequences contributed to the *‘deep-seated discomfort’* – a state of persistent unease with oneself, experienced by the specialists leading to several work-related psychological insecurities. This deep-seated discomfort created a strong enduring sense of distress resulting from underlying unresolved emotions and profoundly impacting their emotional and mental well-being. Ultimately, it became a critical determinant in their decision to remain within or exit the system.

### 3. Action-interaction strategy

Actions and interactions make up the second section of the paradigm model and refer to people’s actual meanings of events in their lives [34].

In Fig 2, the categories *‘decision to quit’* and *‘decision to continue’* represent the action–interaction strategies used by the specialists to cope with their unsatisfactory experiences and the psychological discomfort they developed from working in rural professional settings.

#### 3.1 Action-interaction strategy for those who exit the system.

The decision to quit the healthcare system depicts the *‘risk-taking perspective’* of the specialists who wished to guard their professional identity. This approach indicates the action-interaction strategy of those specialists who left the rural health system and joined private health facilities to protect their professional identity. For many specialists, compromised work autonomy and lack of workplace infrastructure limited their scope of practice to function efficiently. They could not identify themselves with their professional roles which made them feel hopeless about their current aspirations and future career avenues. They felt a strong compulsion to preserve their professional skills, attain higher fiscal benefits and attract favorable professional growth opportunities. Basing their decisions on their personal and family’s economic and psychosocial well-being and leaving the health system to regain their collective sense of self, some specialists found it challenging yet felt compelled to exit the rural healthcare system.

According to a *specialist physician (female, 38 years, rural CHC),*

*‘The biggest issue is that we are educated and learn the medical sciences but do not have the environment to execute our learning there. What is the point of staying there when we have invested ten to twelve years in learning the skills, and despite that, we are not sure about our place in the system!’*

Specialists leaving the system faced conflicts related to professional identity, diminished work autonomy, lowered self-esteem, emotional distress, economic and psychosocial difficulties, risks associated with career transition and challenges sustaining professional competencies, all of which obstructed their professional growth and aspirations.

#### 3.2 Action-interaction strategy for those who remain in the system.

*‘Acceptance and Adaptation’* constituted the action-interaction strategies adopted by the specialists to help them remain continuously committed to their jobs and stay in the system. These strategies reflected the specialists’ adaptive choices to sustain themselves in the challenging environment. Such strategies involved accepting one’s circumstances and accordingly adapting to the existing healthcare system’s functionality while compromising one’s expectations and self-needs. Specialists who remained in the system accepted and adapted to their surroundings drawing strength from their strong spiritual beliefs and philanthropic inclinations toward life and other human beings. They perceived rural people as denied essential healthcare treatment services. They were also compelled by an innate desire to help them to the best possible extent, prioritizing rural service over commercial ambition.

*A specialist physician (male, 32 years, rural CHC) shared,*

*‘See, I have been a spiritual person since the very beginning of my life. So, seeing people’s problems, pains and agonies always used to melt my heart and push me to help them to the best of my capabilities.’*

*Another specialist (male, 38 years, rural CHC) said, ‘I wanted to serve the community from where I belong, and my motive has never been to make it a commercial job. So, despite all my disadvantages and limitations, I have accepted the circumstances and compromised with my expectations of being competitive.’*

Other specialists felt the loss associated with quitting was too high considering the limited comparable job alternatives and the struggle to find one that aligned their professional dignity and economic well-being. They continued working there despite their knowledge, skills and expectations being compromised because of the decent salary packages, incentives for procuring admission to PG courses and the security of a government job. The advantages of government employment outweighed the sacrifices for autonomy, skill enhancement and career advancement.

According to *a PG medical resident (male, 31 years, Government medical college), ‘I never wanted to work in the rural area, but I needed incentives to get admission to PG as it is a very tough exam. Also, sometimes, a government job provides job security and stability.’*

The personal impact of sustained engagement in the rural healthcare system involved sacrifices to one’s professional autonomy and self-esteem, reduced clinical knowledge and the lack of skill development needed for professional growth among specialists.

Both strategies emphasized specialists’ resilience while highlighting the structural adjustments necessary to empower them in their professional roles. Their decision to stay or withdraw from the rural healthcare system has had various repercussions as outlined below.

### 4. Healthcare system consequences

*‘Demotivation and acute shortage of specialist physicians in rural CHCs’* represent the healthcare system consequences of specialists’ decisions to leave or stay in the rural healthcare system. Despite certification as skilled health experts, the specialist physicians stated that they could not offer the treatment they wanted due to the multiple systemic deficiencies (inadequate working conditions, compromised work autonomy and the involvement of non-technical people in their work). Such events were perceived as a significant barrier to their virtuosity in positively influencing their patients’ lives, contributing to their higher attrition rates.

A *specialist physician (male, 37 years, Government DH)* stated, *‘Even if we want to help the patients, we cannot! We can only provide the treatment as per the allotted infrastructure and medication at the rural health centers. It feels helpless not to help somebody who needs our help, so it is better to leave such a system.’*

Specialists further perceived their higher vacancies added to undesirable loops, exacerbating the work pressure for the surviving specialist physicians. The scarcity of qualified specialists in rural CHCs compelled general physicians to undertake responsibilities outside their expertise in addition to the other workplace challenges at the rural health centers. The higher attrition rates of specialists in the system and inadequate working conditions compromised access to standard healthcare facilities. This further led to dissatisfaction among the rural communities, forcing the beneficiaries to seek treatment from higher health facilities.

A *specialist physician (male, 33 years, Government DH)* shared, *‘Due to the shortage of specialists in the rural CHCs, the work pressure is twice, and that too in the absence of proper working conditions. So, specialists are left with no option but to work with whatever is in the system, which makes patients unhappy. Then they come to us at the DHs.’*

**Challenges for female versus male specialist physicians:** Cultural norms, night shifts and insufficient patient assistance led female specialists to report a greater sacrifice of their autonomy and security than their male counterparts. The female specialists shared that their gender identity overshadowed their professional identity in the eyes of their male colleagues and those of the rural community. Because of stereotypical perceptions and cultural beliefs that women are fragile and professionally incompetent, female specialists were seen as more suited for the role of nurses or midwives than specialist physicians.

A *specialist physician (female, 32 years, rural CHC)* shared, *‘No matter how hard work we do, villagers just consider us nurses and midwives. They do not feel that we are even eligible to become physicians.’*

Men are believed to be more qualified for superior professional roles, such as specialists and more capable of sustaining themselves in challenging environments. According to the female specialists, the fact that they were not taken seriously (despite being as competent as their male colleagues) and felt unsafe and insecure in their work environment affected their self-esteem and professional identity. They also reported feeling vulnerable in their workplace due to unwanted attention from sometimes unruly male villagers.

A *specialist physician (female, 35 years, rural CHC)* highlights this with the following anecdote: *‘One night, a drunk patient came into the health center and started staring at me. I felt so uncomfortable! The most important one is the security issue.’*

Further, as the study’s findings explored, PG medical students favored educational and professional trajectories and superior workplace facilities. They were incentivized by benefits such as preferential admission to specialized programs in return for rural service commitments to accept rural assignments.

As shared by a *PG Medical resident (male, 34 years, Private Medical college), ‘I look forward to becoming an assistant professor in some medical college, but I do not know how that is possible once you enter the rural settings. In medical colleges, we have a proper work infrastructure, good equipment, and all the relevant facilities, which is very different from a scenario in a rural setup. However, PG residents also get job incentives, which help them fulfill their expectations.’*

In-service specialist physicians faced challenges in acquiring substantial practical experience in resource-limited settings. However, they were driven by remuneration while displaying flexibility in their dedication to serving rural communities despite challenging circumstances.

*A specialist physician (male, 32 years, rural CHC)* shared, *‘No doubt there are challenging conditions in the rural regions, but the salary structure is a good incentive to work there. Moreover, helping the community also gives me a sense of contentment.’*

The study model discussed above represents a comprehensive exploration of the experiences of specialist physicians in rural health centers of Rajasthan. Based on the paradigm model [32], it delves into specific sets of conditions (disadvantages of underdevelopment, local political climate, and healthcare system factors), intermediate consequences (external and internal), action-interaction strategies (decision to quit or remain in the system) and the related health system consequences (demotivation and acute shortage of specialist physicians) in the rural healthcare context.

## Discussion

The present study emphasizes the complex experiences of specialist physicians focusing on their struggle to preserve their self-esteem and sustain their professional identity within the rural healthcare system in Rajasthan. By influencing one’s action-interaction in terms of remaining in or leaving the system, certain conditions such as the under-resourced rural infrastructure, local political climate and healthcare system structure are found to have an impact on the specialists’ motivation to work at rural CHCs in Rajasthan leading to their higher attrition rates. The lack of comprehensive development and infrastructural amenities including housing, educational facilities for children, remote job locations and inadequate transportation diminishes specialists’ motivation to work in these regions. The structure of healthcare system and power imbalance between local governing bodies and specialists create complex accountability in an under-resourced rural healthcare setting.

The ‘local political climate’ category identified in the study suggests delegating powers to local governing bodies creating an imbalanced mutual dependency relationship between PRI and specialists. Such a scenario results in specialists relying excessively on PRI and hardly making work-related decisions, hampering their ability to effectively utilize expertise in the rural healthcare system. Certain healthcare system practices such as lack of transparent HR policies, inability to maintain professional upskilling, weak reward-performance linkages and issues related to medico-legal cases aggravate the local political climate and contribute to their struggle to preserve self-esteem and professional identity. The effect of the local political climate in influencing health workers’ motivation to work in rural CHCs has also been highlighted by studies in Nigeria [42], Bangladesh [43], Tajikistan [44], Pakistan [45], Ethiopia [46], Timor Leste [47], Malawi, Mozambique and Tanzania [48]. Similarly, case studies conducted in Cambodia, Afghanistan and Mozambique [49] and several recent political and economic initiatives in India [50] report similar evidence regarding the impact of local governance as an essential variable that motivates health workers and generates sustainable health outcomes.

Additionally, our model revealed specific healthcare system factors such as workplace infrastructure, financial incentives and workload level, as factors valued by specialist physicians when deciding to work in rural regions. The results align with the findings from the various international contexts for diverse categories of health professionals such as OB-GYNs in Nepal [51], physicians in Bangladesh [52], Community Health Volunteers in Kenya [53], final-year students of nursing, midwifery and community health and frontline health workers in Cross River State, Nigeria [54], healthcare personnel in South Africa [55], physicians and nurses in Türkiye [56] and nurses in Tehran, Iran [57]. Likewise, when considering rural job postings in the Philippines [58], physicians give more weight to non-financial factors like being more intrinsically motivated to care for underserved rural communities.

In comparing these findings with other Indian studies, similar job factors were previously identified in various contexts. Similar findings have been observed in other Indian states –Nagaland and Meghalaya [59], Gujarat and Madhya Pradesh [60,61], Odisha [62] and Haryana [63]. The physicians here have also valued incentives like salary, career growth opportunities, better working conditions, good schooling, transparent HR policies for postings and performance evaluation, better housing, favorable health center location, willingness to serve the rural community, appropriate staffing capacity and manageable workload.

As explored in our study, such complex interactions between the local political environment and healthcare system factors lead to specific extrinsic (power struggles with senior administrative officials and local governing bodies, uneasy relations with the patients) and intrinsic (compromising with deskilling, self-role distance) consequences, leading to ‘deep-seated discomfort’ in specialists, which manifests as feelings of frustration and persistent distress. This deep-seated discomfort is an intermediate consequence shaping their actions and interactions to stay and exit the healthcare system. Our findings align with a study conducted among specialists in Punjab, a state in India, which identified future career aspirations, social connections and personal well-being as significant factors influencing job satisfaction. Due to the highly demanding nature of their profession, these factors resulted in feelings of uncertainty and low self-esteem among the specialists in Punjab [64].

Many specialists left the system to protect their self-esteem and professional identity in rural healthcare. Nevertheless, some specialists who stayed in the system willingly embraced their limited independence, lowered self-esteem and professional identity while adjusting to their work circumstances because of their intrinsic motivation and the personal benefits of serving the rural community. Further health system consequences follow leading to their demotivation and a higher attrition rate in the public health system. The high attrition rates of specialists make rural communities dissatisfied with access to lower-quality healthcare services. This necessitates immediate policy attention to address the huge vacancies of specialists in the rural healthcare system.

The study’s findings are in contradiction in the international context in various countries, where health workers preferred conditional scholarships for specialization [65], study leaves after two or three years of employment service [66], implementation of effective supervision techniques, such as optimizing face-to-face meetings and utilizing community-monitoring mechanisms [67] and implementing quotas in medical schools for students from underserved areas [68] as significant incentives to work in rural health centers.

From a gender perspective, the study reveals that female specialists face unique challenges in the rural healthcare system in terms of gender stereotyping, long-distance family lives, lack of supportive work environments and security, all contributing to their poor retention rate at rural CHCs. Such gender-specific challenges underscore the urgent need for tailored initiatives to improve the retention of female specialists. Similar experiences have been described by female specialists in various countries like Timor Leste [47], Mozambique, Sierra Leone, Northern Uganda [69] and Lebanon [70].

Our theory aligns with several well-established models of motivation for health workers, such as Hackman and Oldham’s Job Characteristics Model [71], Self-Determination Theory (SDT) [72] and Job-Demands resources (JD-R) model [73], each emphasizing different aspects of what drives healthcare professionals. Hackman and Oldham’s Job Characteristics Model focuses on job design and its impact on employee motivation by identifying five core job dimensions: skill variety, task identity, task significance, autonomy and feedback [71]. Our model’s categories like health system factors (work conditions) and local political climate (including performance feedback and professional upskilling) align with these dimensions highlighting job design’s importance in motivating specialists. Likewise, SDT emphasizes the role of intrinsic motivation and the need for autonomy, competence, and relatedness [72]. Intrinsic motivators, as revealed in our model, such as the desire to serve the community and maintain professional competence, resonate with SDT’s principles. Likewise, the JD-R model posits that high job demands requiring continuous physical or psychological effort can lead to stress and burnout by depleting mental and physical resources [73]. Our model effectively corresponds with the JD-R model by recognizing and addressing the principal job demands and resources discussed as various categories in the model that affect the motivation and retention of specialist physicians in rural healthcare environments.

By integrating and linking various categories around the core category *‘The struggle of preserving Self-esteem and Professional Identity’,* the present study builds a grounded theory of experiences of specialists in rural areas, where the ones who were unable to preserve their self-esteem and professional identity exit the system, while the others who compromised with it remained in the system. The study model offers rich contextual insights into the factors identified from the experiences of specialists, by providing a holistic perspective through interconnecting the socio-political, organizational and personal factors, as instrumental in shaping their motivation. This is essential for addressing their huge shortage rates and developing effective evidence-based interventions. This comprehensive investigation emphasizes how the incapacity of specialists to perform their tasks proficiently affects their self-esteem, professional identity and work drive. It also offers a unique contribution by highlighting the psychological and emotional challenges faced by specialists in rural CHCs. This perspective improves job satisfaction and turnover understanding beyond traditional motivational frameworks by linking theoretical concepts with actual applications.

The study’s findings demonstrate that health system should consider packaging interventions to the specific setting where specialists operate rather than merely offering a standard set of incentive packages given the various conditions influencing specialists’ retention, as explored in the study. In the past, Southeast Asian countries such as Thailand, Bhutan, and the three districts of Chhattisgarh state in India—Dantewada, Sukma and Bijapur—have positively influenced the availability of health personnel and service utilization through the implementation of various rural retention policy packages [74,75]. Therefore, immediate policy attention is imperative to develop appropriate HR strategies and incentive packages to strengthen the morale, accessibility and availability of specialists in the rural healthcare system. Since the health sector cannot make these improvements independently, the cooperation of the finance department, general administration department and other relevant functionaries might be needed to reframe the current policy systems and effectively implement them. Additionally, combined efforts from the federal and state governments are needed to involve key stakeholders in making policies pertinent to specialist physicians’ personal, professional, emotional and social needs when working at rural CHCs of Rajasthan.

### Policy implications

The research provides various evidence-based policy interventions to enhance motivation and mitigate the significant shortage of specialists in rural CHCs of Rajasthan. Policymakers may substantially affect specialist physicians’ decisions to stay in rural areas by improving the rural developmental infrastructure, healthcare system processes and providing a conducive working environment. This can increase job satisfaction and mitigate specialists’ deskilling outcomes. Forming a system of workload-based staffing and periodic clinical rotations could enable specialist physicians to concentrate on their clinical responsibilities. Likewise, introducing career development initiatives, professional networks and public acknowledgment initiatives can help specialist physicians enhance their reputation as dignified health professionals while providing them with the necessary work autonomy. Developing specialist cadres and reframing policies regarding the ownership of PRIs in the healthcare system to improve accountability relationships between the local governing authorities and specialists might develop a sense of professional identity and motivation among the specialists to continue serving in rural areas. The execution of context-specific HR strategies to enhance the retention of specialists, as suggested in the study, aligns with the WHO’s recommendations and findings [76].

### Recommendations

Further research is required to comprehend the larger environment in which specialists work and the impact of the socio-political context on their self-esteem and professional identity in the rural healthcare system. Similarly, further evaluations are needed to generate evidence for designing retention strategies specific to the job factors explored in this study. Merging with quantitative methods like DCE will help enhance retention and job satisfaction among rural specialist physicians.

### Limitations

This study lacks theoretical saturation of themes/categories related to the influence of essential demographic variables, such as participants’ age, gender and family background, as the objective was to identify job indicators for retaining specialists in rural areas using the DCE method. A comprehensive range of categories encompassing the demographic aspects is crucial to understanding the characteristics influencing specialists’ motivation and experiences within the rural healthcare system. Also, the study did not explore the differences in the choices and experiences of PG medical residents from public and private healthcare facilities. It is acknowledged that the characteristics of private medical colleges differ from those of public medical colleges and might offer a more in-depth understanding necessary to examine the experiences of specialists employed in these settings.

## Conclusion

This research assessed and explored specialist physicians’ perspectives and experiences regarding accepting or rejecting rural job postings in Rajasthan (India), using the grounded theory approach by Strauss and Corbin. The constructed paradigm model provided rich insights into the multifaceted interplay of job factors significantly impacting specialists’ self-esteem and professional identity in rural Rajasthan. This interplay ultimately shapes the specialists’ preferences (via different action-interaction strategies) to exit or remain in the rural healthcare system, contributing to their higher attrition rates (healthcare system consequences). The findings indicate that when developing rural retention strategies, more consideration should be given to structuring the rural developmental infrastructure, local governance system and improvement in the healthcare system processes as they influence specialists’ motivation to work in the rural healthcare system. These factors further contribute to various psychological, emotional and social challenges faced by specialists in rural CHCs, leading to their huge vacancies in the rural healthcare system. The state government and key stakeholders should make concerted efforts to implement policies addressing specialists’ personal and professional needs. Developing policies is imperative to reduce their high attrition rates and strengthen the rural healthcare services in Rajasthan.

## Supporting information

S1 Appendix Code list. (XLSX)
